# Axonal tract-integrated finite element brain model for predicting mild traumatic brain injury based on axonal strain

**DOI:** 10.3389/fbioe.2026.1692718

**Published:** 2026-02-27

**Authors:** Noritoshi Atsumi, Yuko Nakahira, Masami Iwamoto

**Affiliations:** Toyota Central R&D Labs., Inc., Nagakute, Aichi, Japan

**Keywords:** axonal fiber tract, axonal injury, brain finite element (FE) model, concussion, MTBI, tractography, traumatic brain injury (TBI)

## Abstract

Mild traumatic brain injury (mTBI), or concussion, is a prevalent public health issue that imposes a substantial economic and social burden on individuals and healthcare systems. Although mTBI is often attributed to brain deformation induced by angular acceleration of the head, its precise mechanisms, including the relationship between local deformations of the brain parenchyma or axonal fibers and clinical symptoms, have not yet been elucidated. Finite element (FE) models of the human brain have been widely used to estimate brain strain in various impact scenarios associated with mTBI. However, despite the possibility that mTBI-related neuropathological changes may involve disruptions of neural pathways connecting different brain regions, most existing models do not account for the architecture of axonal fibers at the anatomical tract level. This study proposes an advanced human brain FE model that explicitly incorporates axonal fiber tracts derived from a group-averaged tractography atlas. The tracts were embedded as a series of continuous beam elements into the solid elements of the brain parenchyma, enabling dynamic evaluation of axonal strain for each tract during head impacts. The model was validated against experimental data on brain deformation from postmortem human subject tests and exhibited comparable or superior correlation scores relative to existing models. Reconstruction simulations of eight real-world mTBI cases, including both vehicular- and sports-related impacts, were conducted using the developed model by comparing two axonal strain-based injury metrics across the cases. The results consistently showed higher injury metrics in specific tract, including the posterior thalamic radiation, body of the corpus callosum, frontal aslant tract, posterior corticostriatal tract, medial lemniscus, parietal corticopontine tract, and superior longitudinal fasciculus. Additionally, horizontal head rotation contributed more significantly to tract-level injury metrics than coronal or sagittal rotation. These findings provide new biomechanical insight into the relationship between mTBI and tract-level axonal damage. Furthermore, the proposed brain FE model may serve as a foundation for understanding mTBI mechanisms from a structural and functional connectome perspective, as a step toward improved mTBI prediction.

## 1 Introduction

Traumatic brain injury (TBI) is a major public health issue affecting both individual health and society (Centers for Disease Control and Prevention, 2021; Yan et al., 2025). The resulting disabilities impose a substantial economic burden on patients, public health systems, and society (Guan et al., 2023). In the United States, approximately 224,000 TBI-related hospitalizations and 61,000 deaths were reported in 2017 (Centers for Disease Control and Prevention, 2021). Globally in 2019, TBI had 27.16 million new cases, 48.99 million prevalent cases, and 7.08 million years lived with disabilities (Guan et al., 2023). In particular, mild TBI (mTBI), also known as concussion, accounts for approximately 81% of TBI incidents (Dewan et al., 2018). The total estimated annual healthcare costs for low-severity TBI in the first year after injury are substantially higher than those for middle- or high-severity TBI (Miller et al., 2021). mTBI can result from various causes, including falls, vehicular accidents, and collisions in sports-related activities associated with angular acceleration of the head (Smith et al., 2000; Shinoda and Asano, 2013; Narayana, 2017). However, the precise mTBI mechanisms that relate local deformation of brain tissue or axonal fibers induced by head rotation with the resulting symptoms remain incompletely understood. A better understanding of these mechanisms is essential for elucidating the pathophysiology of mTBI, developing brain injury criteria, and designing effective head-protective devices.

Finite element (FE) analysis is a potential tool for predicting brain tissue deformation related to mTBI during head impacts. Several FE models of the human brain have been developed and continually updated in the past decades (Kleiven, 2007; Mao et al., 2013; Sahoo et al., 2014; Li et al., 2021; Zhao and Ji, 2020; Zhou et al., 2020; Atsumi et al., 2021; Lyu et al., 2022; Wu et al., 2019). In studies utilizing these brain FE models, maximum principal strain (MPS) (Kleiven, 2007; Giordano et al., 2014; Zhou et al., 2019a; 2020; Atsumi et al., 2018; 2021; Lyu et al., 2022; Gabler et al., 2019) and cumulative strain damage measure (CSDM) (Kleiven, 2007; Takhounts et al., 2008; Atsumi et al., 2016a; 2018; Gabler et al., 2018) have widely been used as strain-based metrics for TBI prediction and development of injury risk functions. However, strain in the direction of axonal fibers (axonal strain) is a better predictor of mTBI than MPS, as reported by (Giordano and Kleiven, 2014b; Zhao et al., 2017; Zhou et al., 2021b). Zhao et al. (2017) proposed an approach for statically associating the axonal strain, which is calculated from the strain tensor of solid elements in the white matter using a brain FE model, with sampling points on axonal fibers extracted from tractography. However, their post-processing approach cannot address dynamic changes in axonal direction because of deformation in the brain parenchyma during head impact, affecting the accuracy of injury prediction (Zhou et al., 2021a). In addition, Zhou et al. (2022) emphasized the importance of integrating neuroimaging-derived fiber orientation into brain FE models without spatial downsampling, as this may lead to deviations in the computation of axonal strain. Therefore, modeling axonal fibers explicitly within the solid elements of the brain parenchyma and dynamically evaluating axonal strain can enhance the biomechanical prediction of mTBI. Wu et al. (2019) proposed an approach for explicitly incorporating white matter axonal fibers into the brain FE model of the Global Human Body Model Consortium (GHBMC) using 1D cable elements. However, because their model was validated only for relative displacements of the brain parenchyma with respect to the skull (Hardy et al., 2001; Alshareef et al., 2021), its accuracy in predicting brain strain under head impact remains unclear.

Previous studies using brain FE models have typically analyzed the degree of deformation or injury risk based on the anatomical region of interest (ROI). For example, Viano et al. (2005) indicated that large strains occur in the fornix, midbrain, and corpus callosum, based on reconstructions of head impacts sustained by athletes in the National Football League using a brain FE model. Reconstruction simulations of rear-end collisions Atsumi et al. (2020) also showed higher MPS values in the midbrain than in the corpus callosum. Zimmerman et al. (2022) demonstrated that head impacts leading to loss of consciousness (LoC) result in higher strain and strain-rates in many brain regions, including the brainstem and cerebellum, and dystonic posturing is associated with increased deformation in cortical regions, including the motor cortex. In addition, the CSDM depends on the cumulative ratio of elements that exceed the threshold value of the MPS with respect to the entire cerebrum. However, several clinical studies based on neuroimaging have demonstrated a relationship between mTBI and the structural/functional connectome of the brain (Papini et al., 2024; Mitra et al., 2016; Rowland et al., 2024; Woodrow et al., 2023; Pandit et al., 2013). For example, Pandit et al. (2013) observed that white matter microstructural damage following TBI could affect the functioning of distributed networks underlying cognitive function through the graph theoretical analysis of functional magnetic resonance imaging data. Woodrow et al. (2023) reported acute thalamic hyperconnectivity in mTBI, as a basis of chronic post-concussive symptoms, despite the absence of structural changes in MRI. A scoping review of altered white matter organization in patients with mTBI conducted by Papini et al. (2024) indicated that brain structures in which diffusion changes are consistently observed include the corpus callosum genu, splenium, and body, internal capsule, corona radiata, fronto-occipital fasciculus, and superior longitudinal fasciculus. These studies showed that mTBI-related neuropathological changes may not be limited to specific anatomical regions but may involve disruptions of neural pathways that interconnect distinct brain regions. Therefore, a biomechanical analysis of mTBI that incorporates axonal fiber tracts is essential for predicting mTBI in relation to changes in brain function and clinical symptoms. By integrating the aforementioned brain FE model incorporating axonal fibers (Wu et al., 2019) with the brain dynamics model based on the Kuramoto oscillator (Kuramoto, 1975), Wu et al. (2022a) observed that injurious head impacts cause significant alterations in the global network topology. Kraft et al. (2012) also proposed an integrative approach based on graph theoretical analysis that relates localized mechanical brain damage, including elevated axonal strain, predicted by FE simulations to degradation of the structural connectome, thereby capturing the spatiotemporal characteristics of trauma. However, the biomechanical mechanisms that contribute to increased axonal strain in specific neural pathways associated with mTBI remain unclear.

This study aimed to identify axonal fiber tracts that are potentially associated with the occurrence of mTBI under real-world head impacts by quantifying axonal strain at the tract level. To this end, we developed a human brain FE model that integrates axonal fiber tracts, a nonlinear constitutive model of the brain parenchyma, and intraventricular cerebrospinal fluid dynamics. Axonal fiber tracts extracted from the group-averaged tractography atlas (Yeh, 2022) were explicitly implemented in a previously developed brain FE model. The developed model was validated against experimental data of postmortem human subjects (PMHSs) on brain deformation during head impact. Using this model, reconstruction simulations based on real-world mTBI cases, including those due to car crashes and collisions in American football games, were conducted. Then, injury metrics based on the axonal strain for each tract were calculated and compared among the mTBI cases. Further, the effect of the direction of head rotation on axonal strain-based injury metrics for each tract was investigated. Through these analyses, we examined and discussed the possibility of mTBI prediction using a brain FE model incorporating axonal fiber tracts.

## 2 Materials and methods

### 2.1 Baseline human brain FE model

**FIGURE 1:**
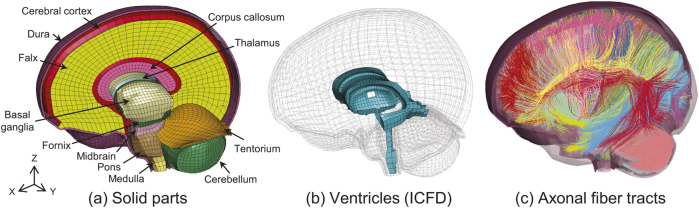
Overview of the brain FE model. **(a)** Parts of solid elements. **(b)** ICFD is applied to the parts of the ventricles and cerebral aqueduct shown in blue. **(c)** Axonal fiber tracts embedded to the solid parts.

In this study, we utilized a previously developed human brain FE model (Atsumi et al., 2021; 2023) and extended it by embedding axonal fiber tracts as streamlines of beam elements into the solid element parts of the brain parenchyma. Figure 1 shows an overview of the proposed model. The model has well-described anatomical structures, including the cerebrum, corpus callosum, thalamus, basal ganglia, fornix, cerebellum, dura mater, falx, tentorium, midbrain, pons, and medulla oblongata using hexahedral solid elements (Figure 1a). The pia mater and arachnoid were modeled using shell elements. The model has 41,000 nodes and comprises approximately 48,000 solid and 13,000 shell elements. The interface between the brain and dura mater was modeled using a layer of solid elements with shared nodes. All simulations in this study were performed using LS-DYNA (LSTC, USA) R13.1.1 MPP in double precision on a workstation equipped with an Intel Xeon Platinum 8358 (2.6 GHz, 32 cores) and 1024 GB of RAM, using 8 CPU cores in parallel.

The material properties of each part of the brain FE model were derived using a previous model (Atsumi et al., 2021; 2023). A nonlinear viscoelastic constitutive model (Atsumi et al., 2016a; 2018) was employed for the brain parenchyma. This model effectively captures the anisotropy in the white matter, strain-rate dependency over a wide range, and characteristic features of the unloading process by formulating a strain energy density function with internal state variables based on a 3D generalized Maxwell model. The anisotropic constitutive model requires the material axes of each solid element of the brain parenchyma to be determined based on the axonal fiber tracts. This is explained in the following section.

To represent the dynamics of the intracranial cerebrospinal fluid (CSF) and the effects of its perfusion pressure on brain deformation, the incompressible fluid dynamics (ICFD) in LS-DYNA (LSTC, USA) is applied to the ventricular part of the model (Figure 1b) (Atsumi et al., 2021). The introduction of ICFD into the intraventricular CSF has been demonstrated in our previous study to improve the validation accuracy of brain strain during head impact (Atsumi et al., 2021). As the boundary of the ventricles was modeled using shell elements as a closed space, the fluid volume meshes inside the ventricles were automatically generated using the ICFD solver in LS-DYNA at the onset of the FE analysis. The boundary condition between the fluid and structure was a non-slip condition, and three boundary layer meshes were defined. Based on the experimental settings of the PMHS test performed in Hardy et al. (2007), an initial pressure of 10.3 kPa was applied to the ICFD part of the model. The fluid–structural interaction was solved in a weak coupling, implying that the solid and fluid solvers proceeded independently with the calculations using individual time steps. The interpolated displacements and forces at the boundary between the structure and fluid were transferred to each other (Livermore Software Technology Corporation LSTC, 2014). The time steps for solving the ICFD and structure were 2.0e-5 and 8.96e-8 s, respectively.

### 2.2 Implementation of axonal fiber tracts

Fiber tracking for mapping axonal pathways requires diffusion MRI data, including diffusion-weighted images (DWIs). In this study, we utilized a group-averaged template preprocessed by Yeh (2022). The template was constructed from DWI dataset (N=1065) of the Human Connectome Project (HCP, https://www.humanconnectome.org/) (Van Essen et al., 2012; Glasser et al., 2016). In the dataset, a multishell diffusion scheme was used. The b-values were 1000, 2000, and 3000 $s/mm^{2}$ ; numbers of diffusion sampling directions were 90, 90, and 90; in-plane resolution was 1.25 mm; and slice thickness was 1.25 mm. Diffusion data were reconstructed in the MNI space using q-space diffeomorphic reconstruction (Yeh and Tseng, 2011) to obtain the spin distribution function (SDF) (Yeh et al., 2010). A diffusion sampling length ratio of 1.7 was used, and the output resolution was 1.0 mm.

The fiber tracking was performed using DSI Studio (https://dsi-studio.labsolver.org/). DSI Studio reconstructs diffusion information in multiple directions within each voxel based on the SDF. Streamlines are tracked by following the corresponding orientation peaks across voxels. This allows tractography to pass through regions with complex configurations, including crossing fibers in the centrum semiovale, rather than collapsing them into a single averaged direction as in conventional tensor-based approaches. An entire brain tractogram was obtained using the group-averaged template with a random seed count of 15,000, maximum length of 300.0 mm, minimum length of 30.0 mm, and step size of 1.0 mm. Then, the tractogram was classified into 84 tracts, including association fibers, projections to the basal ganglia and brainstem, commissural fibers, and cerebellar fibers, based on the tractography atlas proposed by Yeh (2022) using the “Recognize & Cluster” function in DSI Studio. A list of the classified axonal fiber tracts is presented in Table 1. The original tractography atlas (Yeh, 2022) defines neural pathways such as the ansa lenticularis, acoustic radiation, corticobulbar tract, and cranial nerves, including the optic and oculomotor nerves. However, these pathways are excluded from this study because of the limited number of axonal fibers extracted.

**TABLE 1:** List of axonal fiber tracts based on the tractography atlas (Yeh, 2022).

Category	Abbreviation^a^	Name
Association	AF_L/AF_R	Arcuate fasciculus
	C_FPH_L/C_FPH_R	Cingulum, frontal parahippocampal segment
	C_FP_L/C_FP_R	Cingulum, frontal parietal segment
	C_PHP_L/C_PHP_R	Cingulum, parahippocampal parietal segment
	C_PH_L/C_PH_R	Cingulum, parahippocampal segment
	C_PO_L/C_PO_R	Cingulum, parolfactory segment
	EMC_L/EMC_R	Extreme capsule
	FAT_L/FAT_R	Frontal aslant tract
	HA_L/HA_R	Hippocampus alveus
	IFOF_L/IFOF_R	Inferior fronto occipital fasciculus
	ILF_L/ILF_R	Inferior longitudinal fasciculus
	MdLF_L/MdLF_R	Middle longitudinal fasciculus
	TPAT_L/TPAT_R	Temporo-parietal aslant tract
	SLF1_L/SLF1_R	Superior longitudinal fasciculus I
	SLF2_L/SLF2_R	Superior longitudinal fasciculus II
	SLF3_L/SLF3_R	Superior longitudinal fasciculus III
	UF_L/UF_R	Uncinate fasciculus
	VOF_L/VOF_R	Vertical occipital fasciculus
Projection (Basal ganglia)	CS_A_L/CS_A_R	Corticostriatal tract, anterior segment
	CS_S_L/CS_S_R	Corticostriatal tract, superior segment
	CS_P_L/CS_P_R	Corticostriatal tract, posterior segment
	F_L/F_R	Fornix
	OR_L/OR_R	Optic radiation
	TR_A_L/TR_A_R	Thalamic radiation, anterior segment
	TR_P_L/TR_P_R	Thalamic radiation, posterior segment
	TR_S_L/TR_S_R	Thalamic radiation, superior segment
Projection (Brainstem)	CPT_F_L/CPT_F_R	Corticopontine tract, frontal segment
	CPT_P_L/CPT_P_R	Corticopontine tract, parietal segment
	CPT_O_L/CPT_O_R	Corticopontine tract, occipital segment
	CST_L/CST_R	Corticospinal tract
	DRTT_L/DRTT_R	Dentatorubrothalamic tract
	MFB_L/MFB_R	Medial forebrain bundle
	ML_L/ML_R	Medial lemniscus
	NDDT_L/NDDT_R	Non decussating dentatorubrothalamic tract
	RST_L/RST_R	Reticulospinal tract
Commissure	AC_F	Anterior commissure, frontal segment
	AC_O	Anterior commissure, occipital segment
	AC_T	Anterior commissure, temporal segment
	CC_B	Corpus callosum, body
	CC_FMaj	Corpus callosum, forceps major
	CC_FMin	Corpus callosum, forceps minor
	CC_T	Corpus callosum, tapetum
Cerebellum	CB_L/CB_R	Cerebellum
	ICP_L/ICP_R	Inferior cerebellar peduncle
	MCP	Middle cerebellar peduncle
	SCP	Superior cerebellar peduncle
	V	Vermis

^a^ The tracts in the left and right hemispheres are marked with _L and _R at the end.

**FIGURE 2:**
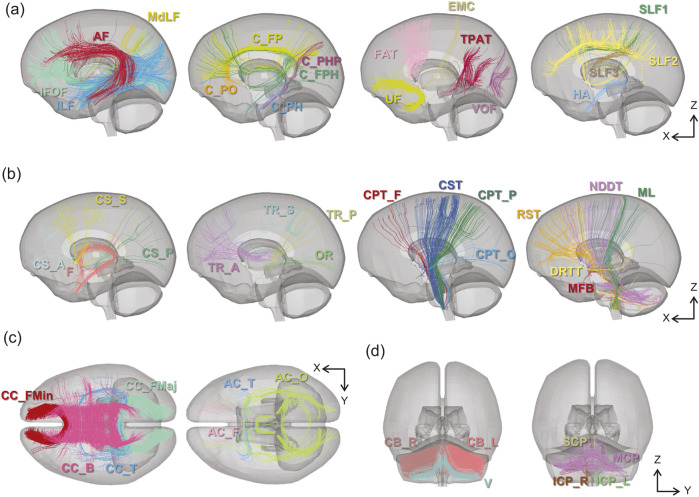
Axonal fiber tracts embedded in the brain FE model in this study based on the tractography atlas proposed by Yeh (2022). Solid elements of the brain parenchyma are displayed as semi-transparent. The nomenclature for each tract is presented in Table 1. **(a)** Association (left) **(b)** Projection (left) **(c)** Commissure **(d)** Cerebellum.

The classified tracts were converted into a format that could be used as beam elements in LS-DYNA using in-house scripts; that is, multiple beam elements were connected to represent a single axonal fiber, and these bundles were represented as axonal tracts. A total of 8,602 axonal fibers, comprising approximately 820,000 beam elements, were ultimately extracted. After aligning the beam elements with the outer shape of the solid parts of the baseline brain FE model using an affine transformation, manual morphing was performed using HyperMesh (Altair, USA) to fit them into the shape of the central region of the brain, including the ventricles. The axonal fiber tracts aligned with respect to the baseline model are shown in Figures 1C, 2 for the entire brain and each tract, respectively. To embed the beam elements into the solid elements of the brain parenchyma for integrated computation without sharing their nodes, the constraint condition keyword *CONSTRAINED_BEAM_IN_SOLID of LS-DYNA was utilized in this study. According to Zhou et al. (2021a), (2022), the material properties of the beam elements were represented using a null model in LS-DYNA, with a mass density and cross-sectional area of 1.0e-11 $ton/mm^{3}$ and 1.0e-11 $mm^{2}$ , respectively. With this modeling approach, the beam elements are effectively ignored in structural analysis and are used solely for computing axonal strain in response to the deformation of the surrounding solid elements. This helps minimize the impact of redundancy in volume or mass between the solid and beam elements, identified as a modeling concern in previous studies (Garimella et al., 2019; Martin et al., 2022).

The material axes of each solid element of the brain parenchyma were determined based on the orientation of the extracted axonal fibers. In this study, the average value of the vectors of the beam elements passing through each solid element was set as the material axis of the solid element using the keyword *ELEMENT_SOLID_ORTHO in LS-DYNA. The starting and ending sides of the extracted axonal fibers could not be distinguished easily. Therefore, although the axial directions of the tracts are similar, they may cancel each other out when averaged, depending on the direction of the vectors. To address this issue, the directions of each vector were unified based on the following equations (Atsumi et al., 2023):

$$v=\left\{\begin{array}{c} v\;\left(prod\left(v\right)\geq 0\right)\\ -v\;\left(prod\left(v\right)<0\right) \end{array}\right.$$ (1)

In Equation 1, the symbol v denotes a 3D vector representing the orientation of a given beam element. The function prod(⋅) computes the scalar product of its vector components.

### 2.3 Validation setup

**FIGURE 3:**
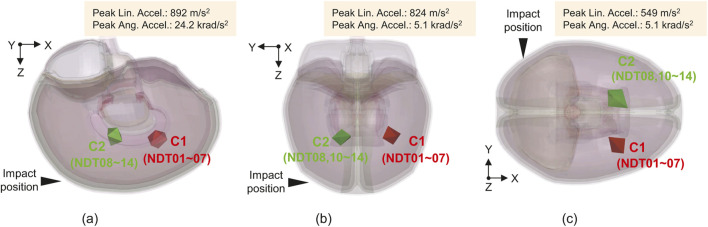
Validation settings for **(a)** C288-T3, **(b)** C380-T1, and **(c)** C380-T2, in which the locations of each cluster (C1 and C2) are indicated (Hardy et al., 2007).

Simulations were performed to validate the proposed tract-embedded model of brain deformation during head impact. The validation protocol was similar to that implemented in previous studies (Atsumi et al., 2021; 2023). PMHS test data on brain deformation during head impacts originally obtained by Hardy et al. (2007) and subsequently reanalyzed with respect to the strain calculation by Zhou et al. (2018), (2019b) were used. Three representative tests were conducted, as shown in Figure 3. C288-T3 is an occipital impact test that results in forward rotation of the head with peak linear and angular accelerations of 892 $m/s^{2}$ and 24.2 $krad/s^{2}$ , respectively (Figure 3a). C380-T1 is a parietal impact test that results in right lateral flexion with peak linear and angular accelerations of 824 $m/s^{2}$ and 5.1 $krad/s^{2}$ , respectively (Figure 3b). C380-T2 is an occipital impact test that results in left rotation of the head with peak linear and angular accelerations of 549 $m/s^{2}$ and 5.1 $krad/s^{2}$ , respectively (Figure 3c). In these experiments, the displacements of each of the seven neural density targets (NDTs) in the two clusters (C1 and C2) embedded in the head of the PMHS with respect to its center of gravity (CG) were measured using a high-speed biplanar X-ray system during head impact (Hardy et al., 2007). Cluster strain was calculated from the relative displacement of the NDTs in each cluster. The experiments were simulated using a cluster model comprising eight tetrahedral elements. First, simulations were performed for each case using the proposed model to calculate the displacements of the nodes closest to each NDT. Subsequently, the history of the displacements of the nodes was input to the corresponding NDT in the cluster model. Finally, the cluster strain was calculated by averaging the Green–Lagrange strain of each element in the cluster model. The four tetrahedral elements in C2 were not included in the cluster strain calculations for C380-T1 and C380-T2 because of the lack of data for the ninth NDT of C2.

The CORrelation and Analysis (CORA) method (Gehre et al., 2009; Gehre and Stahlschmidt, 2011) was used to quantitatively evaluate the validation results of the displacements of NDTs and the strain of each cluster. This method assesses the degree of correlation between a pair of time-history curves using two sub-methods: corridor and correlation methods. However, the former was not activated, and an optimized setting was used, referring to Giordano and Kleiven (2016). The CORA score ranges from 0 to 1, with values closer to 1 indicating better matches. The sliding scales of CORA are defined as follows: “Unacceptable”: 0.0-0.26, “Marginal”: 0.26-0.44, “Fair”: 0.44-0.65, “Good”: 0.65-0.86, and “Excellent”: 0.86-1.0 (The International Organization for Standardization ISO, 1999).

### 2.4 Case study

Reconstruction simulations based on mTBI cases, for which the input curves of head acceleration were available in the literature, were performed using the proposed brain FE model. A summary of the mTBI cases is presented in Table 2. The head acceleration curves for each case are shown in Supplementary Figure S1.

**TABLE 2:** Summary of mTBI cases available from the literature.

Id	Situation	Peak resultant linear accel. [G]	Peak resultant ang. Accel. $\left[rad/s^{2}\right]$	End time [ms]	Acquisition of head accel	Symptoms	Ref.
Case 1	Rear-end collision (ΔV=23km/h)	22.1	1039.0	120	Accident reconstruction by simulation	Headache and neck pain (6 months)	Krafft et al. (1998); Atsumi et al. (2020)
Case 2	American football	106.1	12,950.0	70	Mouthpiece	LoC (2 min)	Hernandez et al. (2015)
Case 3		84.2	6190.0	70		Post-concussive symptoms	Hernandez et al. (2015); Zhou et al. (2021a)
Case 4		96.0	7428.0	100	Video analysis and laboratory reconstruction	Concussion	Sanchez et al. (2019)
Case 5	Side impact (ΔV=27km/h)	27.4	3024.1	140	Accident reconstruction by crash test	LoC^a^	Franklyn et al. (2005)
Case 6	American football	98.8	9117.9	50	Video analysis and laboratory reconstruction	LoC	Zimmerman et al. (2022)
Case 7		82.7	6407.4	50		Dystonic posturing	
Case 8		62.1	3434.9	50		No	

^a^ As it was only declared by the passenger, the reported AIS, was 0 (no injury) (Franklyn et al., 2005).

Case 1 is based on the traffic accident data of a rear-end collision at 23 km/h reported by Krafft et al. (1998). In this case, the driver experienced prolonged headache and neck pain for approximately 6 months following the accident. As the data in Krafft et al. (1998) provided a crash pulse only in the longitudinal direction of the vehicle, we used the acceleration curves of the CG of the head obtained from the reconstruction simulation of the case using a whole-body human FE model proposed in a previous study (Atsumi et al., 2020). As shown in Supplementary Figure S1a, the rotational component around the Y-axis is comparatively large, resulting in neck extension.

Cases 2 and 3 are mTBI incidents from a collegiate American football game reported by Hernandez et al. (2015), where head accelerations were measured using six-degrees-of-freedom (DOF) accelerometers instrumented in mouthpieces. The player in Case 2 experienced an LoC of approximately 2 min after collision, whereas the player in Case 3 did not lose consciousness but reported post-concussive symptoms. Case 2 involved lateral bending of the head toward the right due to a relatively large rotational component around the X-axis (Supplementary Figure S1b), and Case 3 involved forward flexion and lateral bending to the right due to large rotational components around the X- and Y-axes (Supplementary Figure S1c).

Case 4 is an mTBI case from an American football game, as reported by Sanchez et al. (2019). The six-DOF head acceleration curves of the concussed player were derived from laboratory reconstructions using test dummies, based on estimated relative head impact velocities obtained from the video analysis of the collision event. Corrections were made based on an analytical solution of head motion. As shown in Supplementary Figure S1d, the rotational components around the X- and Z-axes were comparatively large, resulting in axial rotation to the right and lateral bending to the left.

Case 5 is based on traffic accident data of a side impact at 27 km/h reported by Franklyn et al. (2005). The head acceleration curves were obtained from a test dummy during the reconstruction of a real-world crash in a full-vehicle crash laboratory. In this case, no codeable head injury of the driver in the target vehicle was originally recorded, that is, AIS 0. However, the first-seat passenger stated that the driver sustained a brief LoC (Franklyn et al., 2005). Thus, in the current study, this case was considered as AIS 2, following a study by Lyu et al. (2022). As shown in Supplementary Figure S1e, the rotational component around the X-axis was large, resulting in lateral bending of the head to the left.

Cases 6, 7, and 8 are based on collision event data from American football reported by Zimmerman et al. (2022). Similar to Case 4, the head acceleration curves in these cases were derived using video analysis and laboratory reconstruction with test dummies. Case 6 is a case with LoC, in which large rotational components around the X- and Y-axes resulted in posterior flexion and lateral bending of the head to the left (Supplementary Figure S1f). In Case 7, the player exhibited dystonic posture. The relatively large rotational components around the X- and Z-axes in Case 7 (Supplementary Figure S1g) resulted in lateral bending to the right and axial rotation to the left. No injury was reported in the player in Case 8, in which a comparatively large rotational component around the X-axis (Supplementary Figure S1h) resulted in lateral bending to the right.

### 2.5 Axonal strain-based injury metrics

The results of the case studies were evaluated using two brain injury metrics based on axonal strain. The first is the enhanced tract-wise injury susceptibility index $\varphi_{tract}^{enh}$ proposed by Zhao et al. (2017). The metric $\varphi_{tract}^{enh}$ is essentially an extension of the concept of CSDM, which was initially developed for the injury evaluation of the entire brain or internal organs modeled using solid elements, to be applied to axonal fiber tracts. $\varphi_{tract}^{enh}$ is defined as follows:

$$\varphi_{tract}^{enh}=\frac{\Sigma w}{\left[\text{Number of axonal fibers in the target tract}\right]},$$ (2)

where w is the weight component corresponding to the damage rate of each axonal fiber in the target tract. The weight w in Equation 2 is defined as follows:

$$w=\frac{\left[\text{Number of beam elements in which axonal strain exceeded}\;\varepsilon_{\text{thresh}}\;\text{in a given axonal fiber}\right]}{\left[\text{Total number of beam elements in a given axonal fiber}\right]}.$$ (3)

The threshold $\varepsilon_{\text{thresh}}$ is set to 0.10, which is an optimal value derived by Zhao et al. (2017). This value is also close to the lower bound of the threshold strain for functional impairment established from *in vivo* tensile tests of adult pig optic nerve by Bain and Meaney (2000). Zhao et al. (2017) derived axonal strain from the strain tensor of a solid element by associating a brain FE model with diffusion tensor images (DTIs). This implies that the weight w in Equation 3 was calculated using axonal strain at the corresponding sampling points on DTI. In this study, as the beam elements corresponding to axonal fibers were explicitly embedded into the solid elements of the brain parenchyma, the axial strain of the embedded beams was used as axonal strain to calculate w .

We used the peak values of the axonal strain in the beam elements of each tract as another injury metric. Following previous studies (Panzer et al., 2012; Sanchez et al., 2019; Zhou et al., 2021a), the 95th percentile value, termed AxS95 in this study, was adopted to limit the effect of numerical instability on the results.

For comparison with the tract-level metrics described above, we also calculated the 95th percentile MPS (MPS95) and CSDM as traditional global injury metrics for all solid elements in the whole-brain (Anderson et al., 2020; Wu et al., 2022a). A threshold of 25% MPS was used to derive CSDM (CSDM25), following previous studies (Wu et al., 2022a; Takhounts et al., 2008; Atsumi et al., 2016b).

## 3 Results

### 3.1 Validation

Tables 3, 4 present the CORA scores for NDT displacement and strain in both clusters, respectively, for each test. Supplementary Figures S2–S7 in the Appendix present details on the history of each NDT and cluster strain. The values listed in Table 3 are the average scores of displacements in the 14 NDTs of both clusters, and those in Table 4 are the average scores of MPS and maximum shear strain in both clusters. For comparison, the reported CORA scores for a detailed and personalizable (ADAPT) head model (Li et al., 2021) are provided in these tables. Notably, only the C1 scores of the ADAPT model for the cluster strain are presented in Table 4 because the C2 results were not available (Li et al., 2021).

**TABLE 3:** Comparisons of average CORA scores for NDT displacement.

Test ID	Current study	ADAPT model (Li et al., 2021)
C288-T3	0.516 (fair)	0.588 (fair)
C380-T1	0.595 (fair)	0.694 (good)
C380-T2	0.522 (fair)	0.549 (fair)

**TABLE 4:** Comparisons of average CORA scores for cluster strain.

Test ID	Current study	ADAPT model (Li et al., 2021)^a^
C288-T3	0.766 (good)	0.742 (good)
C380-T1	0.823 (good)	0.877 (excellent)
C380-T2	0.894 (excellent)	0.793 (good)

^a^ Averaged scores for principal strain and shear strain only from C1.

The CORA scores for NDT displacement were 0.516 for C288-T3, 0.595 for C380-T1, and 0.522 for C380-T2, all of which were rated as “Fair”. The proposed model exhibited slightly lower results in all three tests than the ADAPT model. The CORA scores for cluster strain were 0.766 for C288-T3 and 0.823 for C380-T1, both of which were rated as “Good”. The score for C380-T2 was 0.894, which was rated as “Excellent”. The proposed model yielded lower results for C380-T1, but higher results for C288-T3 and C380-T2 than the ADAPT model.

### 3.2 Case study

**FIGURE 4:**
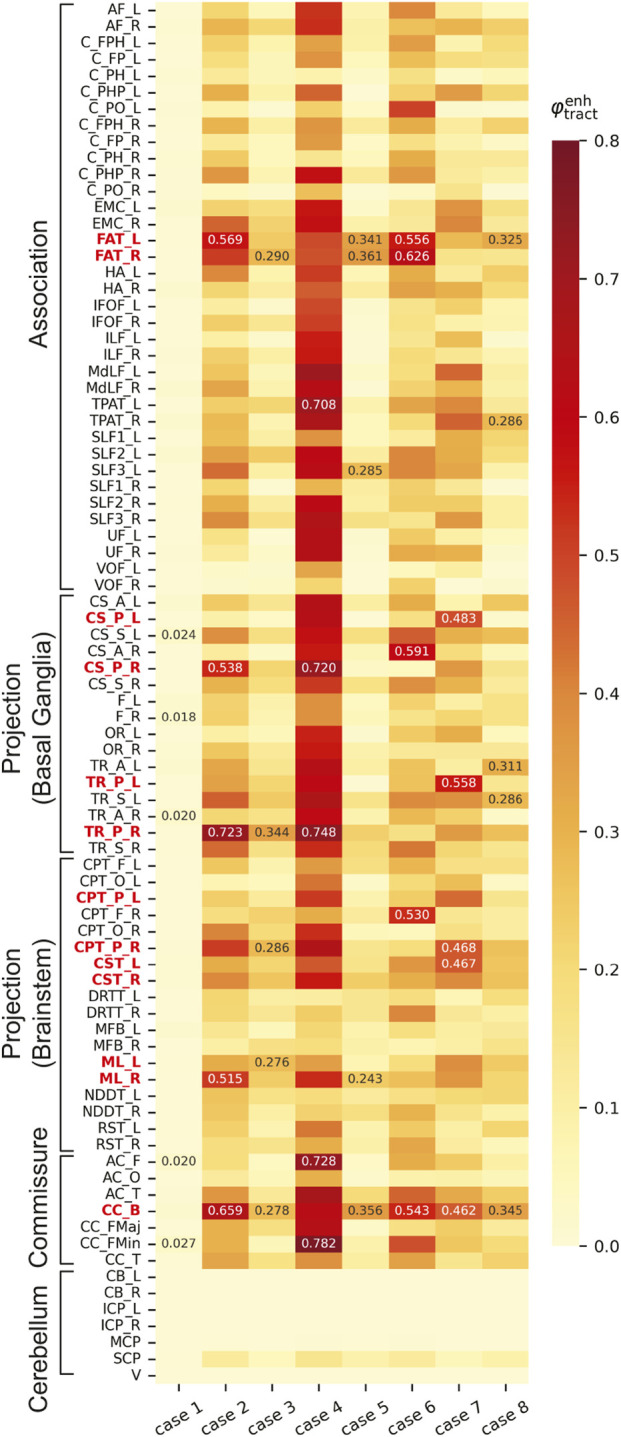
Comparisons of $\varphi_{tract}^{enh}$ for each tract across cases. The nomenclature for each tract is shown in Table 1. The labels of key tracts discussed in the main text (FAT, CS_P, TR_P, CPT_P, CST, ML, and CC_B) are highlighted in bold red.

Figure 4 compares $\varphi_{tract}^{enh}$ for each tract across all cases, where the top five tracts in each case are annotated with numerical values. For Case 1, $\varphi_{tract}^{enh}$ values are low across nearly all tracts. In Case 2, high $\varphi_{tract}^{enh}$ values are observed in the following order: right posterior thalamic radiation (TR_P), body of the corpus callosum (CC_B), left frontal aslant tract (FAT), right posterior corticostriatal tract (CS_P), and right medial lemniscus (ML). In Case 3, the overall values are lower than those in Case 2, with relatively high $\varphi_{tract}^{enh}$ values observed in the right TR_P, right FAT, right parietal corticopontine tract (CPT_P), CC_B, and left ML. Case 4 exhibits the highest values among all cases, particularly in the forceps minor of the corpus callosum (CC_FMin), right TR_P, frontal anterior commissure (AC_F), right CS_P, and left temporo-parietal aslant tract (TPAT). In Case 5, a tendency toward high $\varphi_{tract}^{enh}$ values is observed in the following order: right FAT, CC_B, left FAT, left superior longitudinal fasciculus III (SLF3), and right ML. In Case 6, higher $\varphi_{tract}^{enh}$ values are observed in the right FAT, right anterior corticostriatal tract (CS_A), left FAT, CC_B, and right frontal corticopontine tract (CPT_F). In Case 7, the left TR_P, left CS_P, right CPT_P, left corticospinal tract (CST), and CC_B exhibit high $\varphi_{tract}^{enh}$ values. In Case 8, relatively high $\varphi_{tract}^{enh}$ values are observed for the CC_B, left FAT, left anterior thalamic radiation (TR_A), left superior thalamic radiation (TR_S), and right TPAT; however, the overall values are lower than those in Cases 6 and 7. Cerebellar tracts did not exhibit high $\varphi_{tract}^{enh}$ values in all cases.

**FIGURE 5:**
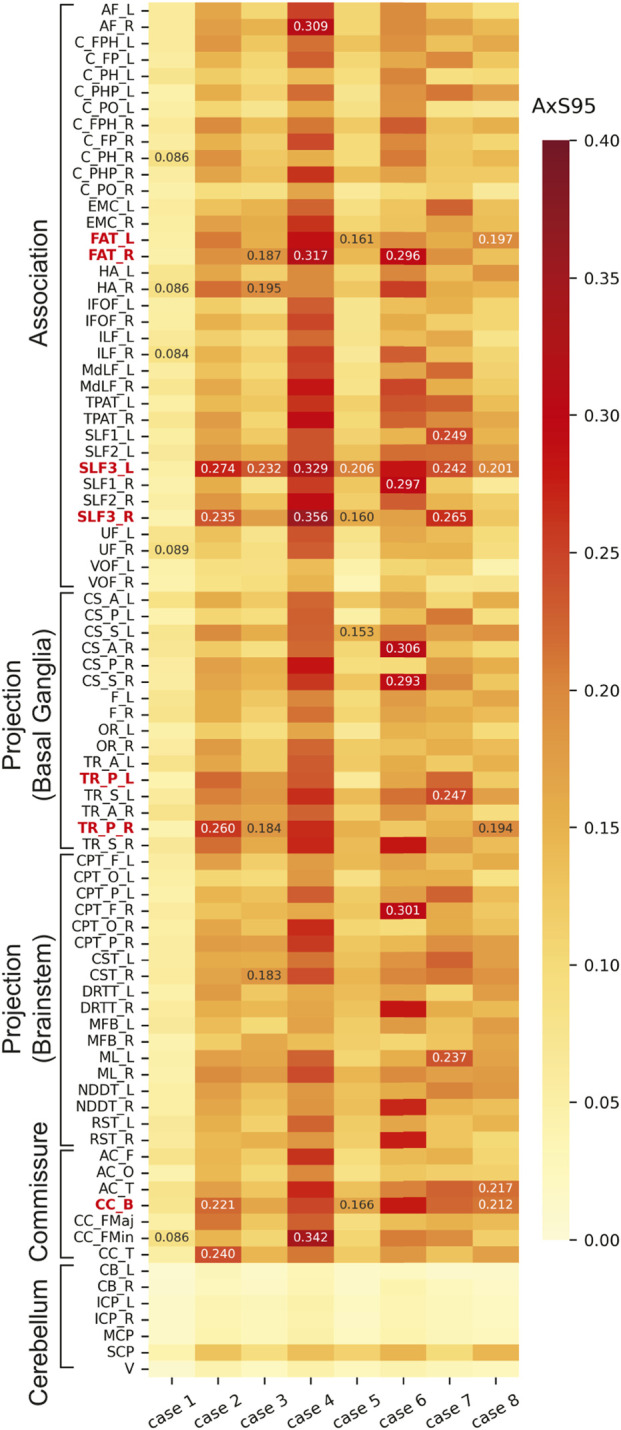
Comparisons of AxS95 for each tract across cases. The nomenclature for each tract is shown in Table 1. The labels of key tracts discussed in the main text (FAT, SLF3, TR_P, and CC_B) are highlighted in bold red.

Figure 5 compares the AxS95 values for each tract in all cases. In Case 1, the AxS95 values are the lowest overall, with a maximum of 0.089 observed in the right uncinate fasciculus (UF). In Case 2, the highest AxS95 values are observed in the left SLF3, right TR_P, tapetum of the corpus callosum (CC_T), right SLF3, and CC_B. In Case 3, the left SLF3 exhibits the highest AxS95 value, followed by the right hippocampus alveus (HA), right FAT, right TR_P, and right CST. Case 4 exhibits the highest AxS95 values among all cases, particularly in the right SLF3, CC_FMin, left SLF3, right FAT, and right arcuate fasciculus (AF). In Case 5, the AxS95 values tend to increase in the following order: left SLF3, CC_B, left FAT, right SLF3, and right superior corticostriatal tract (CS_S). Case 6 exhibits high AxS95 values in the right CS_A, right CPT_F, right superior longitudinal fasciculus I (SLF1), right FAT, and right CS_S, with overall values higher than those in Cases 7 and 8. In Case 7, the highest AxS95 values are observed in the right SLF3, left SLF1, left TR_S, left SLF3, and left ML. In Case 8, high AxS95 values are observed in the temporal anterior commissure (AC_T), CC_B, left SLF3, left FAT, and right TR_P.

To provide a quantitative summary, we examined the association between axonal strain-based injury metrics and reported clinical outcomes using rank-based correlation analysis. Here, clinical outcomes were categorized on a three-level ordinal scale based on the symptom descriptions in the original reports. Specifically, we defined an ordinal outcome category as follows: 0 = no clinical symptoms reported (Case 8); 1 = prolonged/post-concussive symptoms without acute neurological signs (Cases 1 and 3); and 2 = concussion and/or acute neurological signs such as LoC or dystonic posturing (Cases 2, 4, 5, 6, and 7). We then assessed rank correlations between the ordinal outcome category and each injury metric. For each case, we used the mean of the top five tract values across tracts as a representative value for each metric. Spearman’s rank correlation coefficients between the ordinal outcome category and these representative values were ρ=0.784 (p=0.021) for $\varphi_{tract}^{enh}$ and ρ=0.550 (p=0.158) for AxS95. Kendall’s τ showed consistent directions ( τ=0.596 for $\varphi_{tract}^{enh}$ and τ=0.413 for AxS95).

Table 5 compares the peak MPS95 and CSDM25 across all cases. Case 1 shows the lowest values among all cases for both MPS95 and CSDM25. Case 5 exhibits the second lowest MPS95 and CSDM25 values, next to Case 1. Among all cases, MPS95 is highest in Case 6, whereas CSDM25 is highest in Case 4. In addition, the MPS95 in Case 8 is higher than those in Cases 2 and 3, whereas CSDM25 is only slightly lower than in these two cases.

**TABLE 5:** Comparisons of peak MPS95 and CSDM25 across cases.

Id	MPS95	CSDM25 [%]
Case 1	0.149	0.267
Case 2	0.275	14.361
Case 3	0.262	10.817
Case 4	0.387	50.473
Case 5	0.226	3.709
Case 6	0.418	38.738
Case 7	0.305	21.279
Case 8	0.314	10.686

**FIGURE 6:**
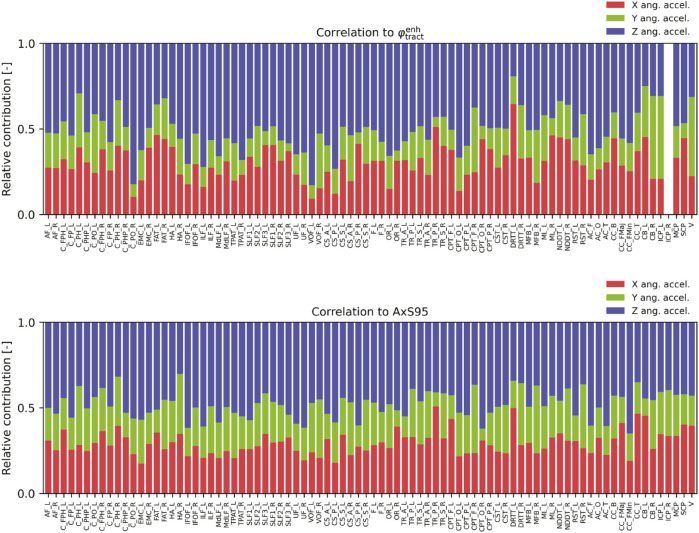
Comparisons of relative contribution of angular accelerations around each axis to injury metrics: $\varphi_{tract}^{enh}$ (upper) and AxS95 (lower). The nomenclature for each tract is presented in Table 1.

Furthermore, the effects of the head rotation direction on the injury metrics for each tract were investigated. Figure 6 compares the contributions of the angular accelerations of the head around each axis to $\varphi_{tract}^{enh}$ and AxS95 across the cases. These are the relative contributions derived by correlating the peak values of the head angular acceleration around the X-, Y-, and Z-axes inputted among cases with injury metrics for each tract (Figures 4, 5). The values in Figure 6 are normalized such that the sum of each axis is 1.0. The bars representing the correlation with $\varphi_{tract}^{enh}$ in the right inferior cerebellar peduncle (ICP) are blanked in the upper part of Figure 6. This is because the values of $\varphi_{tract}^{enh}$ for the right ICP were zero in all cases in this study, and correlation coefficients could not be calculated. Figure 6 shows that the contributions of angular acceleration around the Y-axis are comparatively small for both $\varphi_{tract}^{enh}$ and AxS95, whereas those around the Z-axis are relatively large across most tracts. At the individual tract level, the contributions of angular acceleration around the X-axis to $\varphi_{tract}^{enh}$ were more considerable than those around the Z-axis in the parahippocampal cingulum (C_PH) and FAT, right (TR_P), left dentatorubrothalamic tract, non-decussating dentatorubrothalamic tract, and CC_B. Similarly, compared with those around the Z-axis, the contributions of the angular acceleration around the X-axis were significant for AxS95 in the right C_PH, right TR_P, left CPT_F, left DRRT, and CC_T.

## 4 Discussion

### 4.1 Significance of study

In this study, we investigated tract-specific mechanisms in real-world mTBI cases by evaluating axonal strain-based injury metrics for each tract using an advanced human brain FE model that explicitly incorporates axonal fiber tracts. The baseline model utilized in this study included a nonlinear viscoelastic constitutive model of the brain parenchyma, which comprehensively describes the anisotropy in the white matter, strain-rate dependency over a wide range of strain rates, and characteristics in the unloading process (Atsumi et al., 2016a). Furthermore, axonal fibers extracted from tractography based on SDF (Yeh et al., 2010; Yeh and Tseng, 2011) were embedded as a series of continuous beam elements into the solid elements of the brain parenchyma. Therefore, the developed model can dynamically estimate axonal strain in response to directional changes in axons caused by brain deformation during head impact. Few existing brain FE models employ both a nonlinear constitutive model of the brain parenchyma and axonal fiber tracts obtained using tractography. This integrated modeling approach contributes to the accurate prediction of tract-specific injury risks in the mTBI cases with various impact levels reconstructed in this study. As a result, we identified axonal tracts that consistently exhibited elevated injury risk across multiple mTBI cases (see Section 4.4 for further details). This finding represents a key step toward understanding the mechanisms of mTBI from the perspective of alterations in both the structural (Papini et al., 2024; Mitra et al., 2016) and functional connectome (Rowland et al., 2024; Woodrow et al., 2023; Pandit et al., 2013). The widespread and progressive disruption of sodium channels and nodes of Ranvier on axons could be an important substrate underlying brain network dysfunction after concussion (Song et al., 2022). Therefore, mTBI mechanisms should be further investigated at the level of mechanical deformation level in axonal fibers and in terms of subsequent alterations in axonal excitability and neural signaling.

### 4.2 Comparison with existing brain FE models

Several studies have proposed brain FE models for mTBI prediction, accounting for the arrangement of axonal fibers. Zhou et al. (2022) adopted a method of constraining multiple beam elements representing axonal fiber orientations obtained from DTI to solid elements of the brain parenchyma. However, because they used discretized beam elements corresponding to each DTI voxel, their association with the tractography atlas may not have been considered. Additionally, DTI specifies a single principal fiber direction per voxel, implying that it cannot address regions with complex fiber orientations, such as crossing fibers (Yeh et al., 2010). By contrast, tractography based on SDF (Yeh et al., 2010; Yeh and Tseng, 2011) employed in the current study enables the reconstruction of such intricate axonal fiber configurations, thereby enabling more accurate modeling of axonal pathways.

More comparable modeling approaches can be found in Wu et al. (2019), (2022a). Wu et al. (2019) implemented axonal fibers as a series of beam elements into the brain FE model of GHBMC using the keyword *CONSTRAINED_BEAM_IN_SOLID in LS-DYNA, similar to that used in this study, attempting integration with functional networks of the brain (Wu et al., 2022a). The group-averaged tractography atlas used in their study was based on a previous generation of datasets from HCP (Glasser et al., 2013; Yeh et al., 2018). In addition, the total number of embedded axonal fibers was 3,446, comprising 104,866 beam elements, which was less than half of that of the model developed in this study. Although their study included a viscoelastic constitutive modeling of axonal fibers as beam elements (Wu et al., 2019), the validation of the model was limited to the relative displacements of the brain parenchyma with respect to the skull (Hardy et al., 2001; Alshareef et al., 2021). Therefore, the sufficiency of the prediction accuracy of brain strain in their models was yet to be verified.

### 4.3 Validation performance

As presented in Table 4, the developed brain FE model exceeded the CORA evaluation value reported in the previous study (Li et al., 2021) in two PMHS tests for cluster strain, demonstrating sufficient validation accuracy. The higher CORA scores for cluster strain can mainly be attributed to the improved accuracy in representing local deformations in deep brain regions by using a calibrated constitutive model of brain parenchyma and ICFD within the ventricles. Although numerous brain FE models have been proposed in the past decades and are considered a basis for strain-based mTBI prediction, few models have been validated against brain strain during head impact. Considering that the clusters in the three PMHS tests simulated in this study were located in the central part of the brain, the validation results indicated that the developed model could accurately predict strain in this region. To the best of our knowledge, there are no cadaveric experimental datasets available for validating brain strain outside the deep brain region during injurious head impact. In fact, the constitutive model of the brain parenchyma used in this study has been validated against multiple sets of material test data using specimens acquired from the corona radiata and cerebral cortex over a wide range of strain rates (Atsumi et al., 2018). In addition, the material property of the pia-arachnoid complex (PAC) layer, which effectively contributes to the relative motion between the brain and skull, has been calibrated against shear test data at different strain rates (Atsumi et al., 2021). Therefore, although it remains challenging to directly validate strain distributions in other regions, including superficial cortex, during head impact, the proposed model provides reasonable confidence that it can reproduce brain deformations under injurious loading conditions. Regarding NDT displacements, the CORA values were slightly lower than those of the ADAPT model (Li et al., 2021) in all tests (Table 3). However, the results were within the “Fair” evaluation range and thus considered to be generally acceptable. This difference between the CORA scores for cluster strain and NDT displacements reflects the modeling strategy adopted in this study, in which the material model was carefully calibrated to reproduce local brain deformations rather than to personalize the global head and brain geometry to each PMHS, as in the ADAPT model (Li et al., 2021). As the ADAPT model (Li et al., 2021) was developed as a personalizable model that could match the shape of the head and brain of an individual, the model was validated against the PMHS tests on brain deformation after scaling to match the dimensions of the model to those of PMHS in each test. This could be attributed to the differences in CORA values for NDT displacements between the developed model in this study and the ADAPT model.

### 4.4 Tract-specific analysis in case studies

Comparisons of axonal strain-based injury metrics among all cases indicated that higher values of both $\varphi_{tract}^{enh}$ and AxS95 were observed in Cases 2, 4, and 6, in which the subjects suffered mTBI, as shown in Figures 4, 5. In particular, Case 4 exhibited the highest overall values. As presented in Table 2, the head acceleration level in Case 4 was lower than those in Cases 2 and 6. This aligns with the commonly accepted concept that the risk of mTBI cannot be evaluated only based on the magnitude of the head acceleration (Ji et al., 2022). In addition, the acceleration waveforms in Case 4 exhibited a longer phenomenon duration than those in Cases 2 and 6, with multiple peaks in the angular accelerations (Supplementary Figure S1b, d, f). Our previous study (Atsumi et al., 2016b) also showed that the duration of head impact affects the value of CSDM. Thus, the increased values of $\varphi_{tract}^{enh}$ and AxS95 in Case 4 could have been caused by the sustained and complex input curves of acceleration. However, Case 1, which is an injury case in a rear-end collision, exhibited the lowest values of $\varphi_{tract}^{enh}$ and AxS95 among all cases (Figures 4, 5). The reported symptoms, including headache and neck pain, of the driver in Case 1 could have been caused by neck injuries, such as whiplash, rather than mTBI. Case 5 involved a vehicle accident in which the driver experienced a brief LoC. However, the values of the injury metrics in Case 5 were lower than those in Case 8, despite the latter having no reported mTBI-related symptoms. Although direct comparisons would be challenging because of the differences in the measurement methods of head acceleration and the duration of the phenomenon, the higher $\varphi_{tract}^{enh}$ values in the CC_B and FAT in Case 5 show that the localized damage in these tracts could have contributed to the occurrence of LoC.

Comparisons of traditional global metrics, including MPS95 and CSDM25 (Table 5), indicate that relatively higher values are observed in Cases 4 and 6, in line with the axonal strain-based injury metrics. By contrast, Case 2, despite the observation of a brief LoC, exhibits an MPS95 value lower than that in Case 8, in which no clinical symptoms were reported. In addition, the differences in CSDM25 values among Cases 2, 3 (post-concussive symptoms), and 8 are relatively small. These findings suggest that conventional global injury metrics may not fully capture the differences in symptoms observed in these cases. The axonal strain-based injury metrics used in this study provide a means to investigate the vulnerabilities specific to particular tracts and their relationships with clinical symptoms that cannot be captured by global metrics.

**FIGURE 7:**
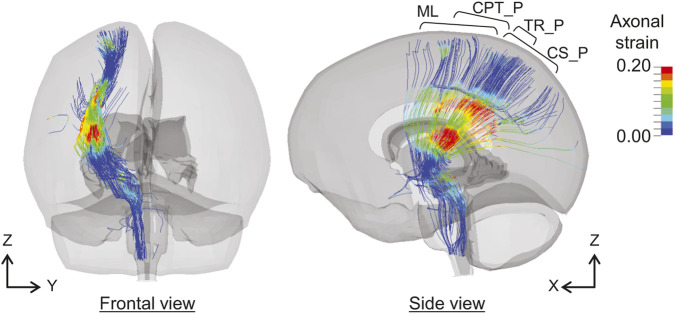
Distributions of axonal strain in the right TR_P, CS_P, ML, and CPT_P at 24 ms in case 2. TR_P, posterior thalamic radiation; CS_P, posterior corticostriatal tract; ML, medial lemniscus; CPT_P, parietal corticopontine tract.

As shown in Figure 4, comparatively higher values of $\varphi_{tract}^{enh}$ are predicted in tracts, including posterior thalamic radiation (TR_P), body of the corpus callosum (CC_B), frontal aslant tract (FAT), posterior corticostriatal tract (CS_P), medial lemniscus (ML), and parietal corticopontine tract (CPT_P), in Cases 2–7, in which mTBI or related symptoms are observed. Figure 7 shows the distributions of the axonal strains of the right TR_P, CS_P, ML, and CPT_P at 24 ms in Case 2. These axonal fiber tracts project to the cerebral cortex of the parietal lobe from the central part of the brain or brainstem, passing near the lateral ventricles. As shown in Figure 7, a higher axonal strain is observed around the ventricles. The higher $\varphi_{tract}^{enh}$ values in TR_P, CS_P, ML, and CPT_P can be attributed to the increased axonal strain in these regions, which is caused by the perfusion pressure represented by ICFD in the ventricles.

**FIGURE 8:**
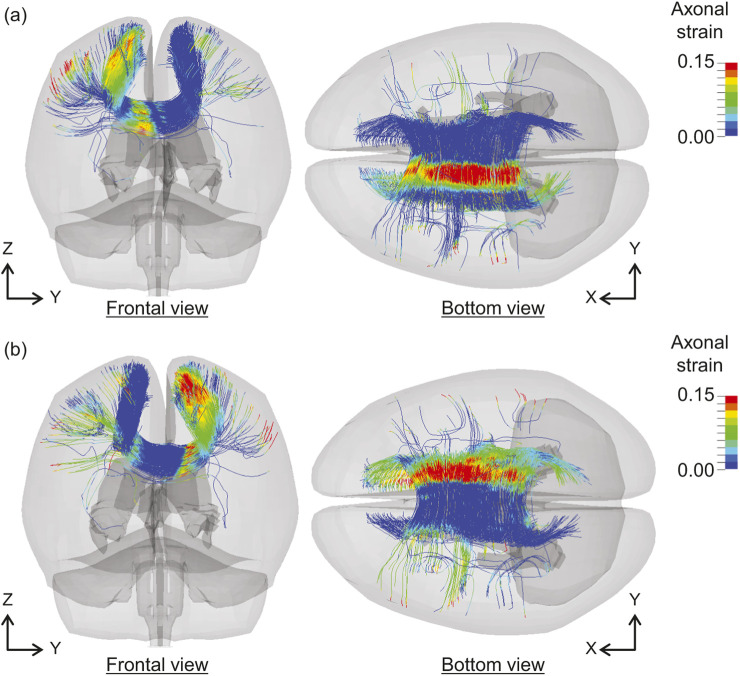
Distributions of axonal strain in CC_B at **(a)** 52 ms and **(b)** 84 ms in Case 5. CC_B, body of corpus callosum.

Figure 8 shows the distributions of the axonal strains of the body of the corpus callosum (CC_B) at (a) 52 and (b) 84 ms for Case 5. A higher axonal strain is observed in the region connecting both hemispheres over the lateral ventricles and close to the medial surface of the cerebral hemispheres. Furthermore, the angular acceleration curves of the head (Supplementary Figure S1E) show that the axonal strain increased on both sides at approximately 52 and 84 ms, corresponding to the negative and positive peaks of the angular accelerations at 40 and 65 ms, respectively. Thus, widespread increments in axonal strain could cause a higher value of $\varphi_{tract}^{enh}$ in CC_B. Such temporally and regionally modulated strain responses in CC_B may be effectively captured by the nonlinear viscoelastic constitutive model employed in this study (Atsumi et al., 2016a), accounting for the characteristic features of the brain parenchyma during the unloading process. Based on statistical analysis of neuroimages of patients with TBI (Asano et al., 2012; Nakayama et al., 2006) and the reports of existing brain FE models (Viano et al., 2005; Zhao et al., 2017; Atsumi et al., 2018), the corpus callosum has been identified as a vulnerable region. These results are consistent with those of the present study.

**FIGURE 9:**
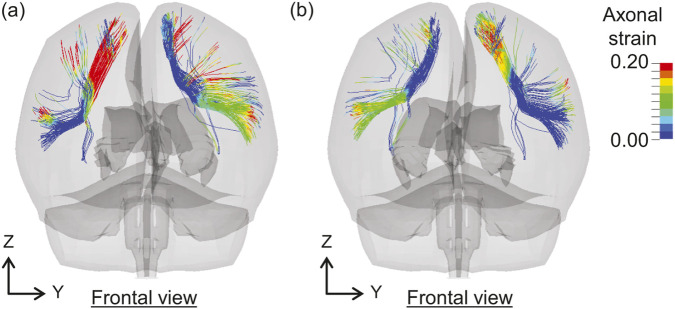
Distributions of axonal strain in the left and right FAT at **(a)** 22 ms and **(b)** 36 ms in Case 6. FAT, frontal aslant tract.

The frontal aslant tract (FAT) is a tract of association fibers connecting the superior to the inferior frontal gyrus in the frontal lobe. Figure 9 shows the axonal strain distributions in the left and right FATs at (a) 22 and (b) 36 ms in Case 6, respectively. In contrast to TR_P or CS_P, as shown in Figure 7, a higher axonal strain was observed in FAT near the cerebral cortex than around the ventricles. These regions close to the cerebral cortex are distant from the CG of the head and are therefore susceptible to shear forces caused by head rotation. This could account for the increased $\varphi_{tract}^{enh}$ values of FAT on both sides.

**FIGURE 10:**
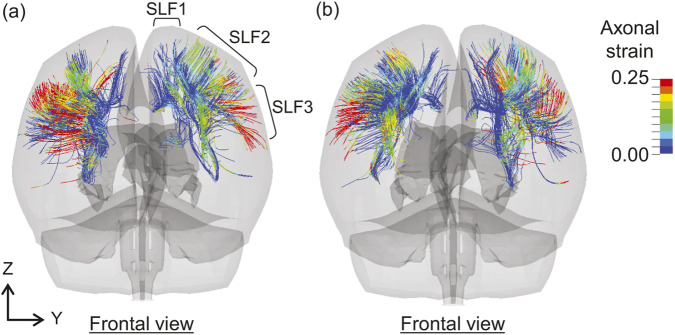
Distributions of axonal strain in the SLF of both hemispheres at **(a)** 20 ms and **(b)** 64 ms in Case 4. SLF, superior longitudinal fasciculus.

For AxS95, high values were observed in TR_P, the corpus callosum, including CC_B and CC_FMin, and FAT, except in Case 1 (Figure 5). Furthermore, the left and right superior longitudinal fasciculus (SLF), particularly SLF3, exhibited high AxS95 values. The SLF is a tract of association fibers connecting the cerebral cortex in the anterior–posterior direction in each hemisphere and is classified as SLF1, SLF2, and SLF3 from the dorsal to the ventral region of the cerebrum. Figure 10 shows the axonal strain distributions in the SLF of both hemispheres at (a) 20 and (b) 64 ms in Case 4. Figure 10 shows that the axonal strain in SLF3 is higher in regions closer to the cerebral cortex than in the central part of the brain. This could be attributed to their susceptibility to shear forces, similar to FAT. Using a brain FE model, Zhao et al. (2017) indicated that the most vulnerable tracts include SLF, anterior limb of the internal capsule, and corpus callosum. Considering that the internal capsule includes the thalamic radiation and corticopontine tract, our findings on the estimated injury metrics for each tract are generally consistent with those of previous studies.

In Case 7, the struck player exhibited dystonic posturing (Zimmerman et al., 2022). A characteristic of the calculated injury metrics in Case 7 is the relatively higher value of $\varphi_{tract}^{enh}$ in corticospinal tract (CST) than those of the other cases. The CST, which is a descending tract from the cerebral cortex to the spinal cord, contributes to the control of voluntary movements. Thus, axonal damage to this tract may induce a dystonic posture. Zimmerman et al. (2022) observed that players who exhibited a dystonic posture after collision had significantly high MPS values in motor regions including the cortical motor area and left CST. This finding is consistent with the results in this study.

The rank-correlation analysis showed a moderate-to-strong association between axonal strain-based injury metrics and the ordinal clinical outcome category (Spearman’s ρ=0.784 for $\varphi_{tract}^{enh}$ and ρ=0.550 for AxS95). However, these findings should currently be interpreted as exploratory. The present dataset of mTBI cases is small, and only one case has no reported symptoms. In addition, the reported clinical outcomes vary in type and level of detail across publications, making it difficult to convert them into a standardized severity scale. For example, Case 4 was described as concussion without explicit reporting of LoC. When Case 4 was alternatively classified as level 1 rather than level 2, Spearman’s ρ decreased to 0.443 for $\varphi_{tract}^{enh}$ and 0.209 for AxS95, suggesting uncertainty in effect size estimates and the need for larger datasets with standardized clinical outcome measures.

### 4.5 Effect of head rotational direction

The effect of head rotational direction on the injury metrics varies depending on the axonal fiber tracts, as shown in Figure 6. However, the overall trend indicated that sagittal rotation around the Y-axis had the least contribution to $\varphi_{tract}^{enh}$ and AxS95, whereas horizontal rotation around the Z-axis had the highest contribution. Wu et al. (2022a), Wu et al. (2022b) investigated the effects of head size and rotational direction on brain functional impairment by integrating a brain FE model with a Kuramoto oscillator model (Kuramoto, 1975), associating the structural and functional networks of the brain. They estimated a higher risk of injury due to rotation in the horizontal plane than in the coronal or sagittal plane. Similarly, Carlsen et al. (2021) demonstrated, through comprehensive analyses using a 2D brain FE model, that horizontal head rotations induce the largest brain tissue strain and strain-rate, followed by coronal and then sagittal rotations. Moreover, Smith et al. (2000) found that immediate and persistent coma related to diffusion axonal injury in miniature swine is consistently produced by axial-plane rotation of the head, rather than by coronal-plane rotation. The findings of the present study are consistent with those of previous studies.

### 4.6 Limitations and future directions

This study had the following limitations. First, although the developed brain FE model included the principal direction of anisotropy of the white matter based on the beam elements within each solid element, the density distribution of the axonal fibers was not considered. Constitutive models of the brain parenchyma in previous studies (Sahoo et al., 2014; Giordano and Kleiven, 2014a; Zhou et al., 2022) have incorporated parameters reflecting fractional anisotropy (FA) values derived from DTI, enabling the spatial representation of anisotropy strength in the white matter. If such fiber density distributions or FA-weighted anisotropy were incorporated, the material properties in regions with lower fiber density or with crossing or branching fibers would become more isotropic. This would imply a reduction in stiffness along the primary axonal fiber direction. Consequently, axonal strain would be expected to become relatively higher in these regions. This could lead to greater variability in axonal strain within each tract and more localized strain concentrations than those obtained in this study using a constitutive model with a uniform strength of anisotropy (Figures 7–10). As the constitutive model of the brain parenchyma used in this study was validated against multiple material tests on anisotropy and strain-rate dependency (Atsumi et al., 2016a; 2018), the prediction accuracy of the current model is comparable to that of previous studies. The introduction of parameters for computing the strength anisotropy is expected to further improve the brain FE model, particularly in the development of subject-specific models associated with individual neuroimaging data. Furthermore, Reiter et al. (2025) revealed that the alignment of axons, glial cells, and blood vessels varies across brain regions, contributing to regional differences in the mechanical properties of the brain parenchyma. Therefore, future models should incorporate region-specific mechanical properties based on these microstructural components.

Second, the classification of axonal fiber tracts used in this study was based on an anatomically identified group-averaged atlas (Yeh, 2022). Considering the relationships between clinical symptoms in mTBI and changes in the functional connectivity of the brain (Pandit et al., 2013; Woodrow et al., 2023), linking structural damage in specific tracts to disruptions in functional networks may provide deeper insights into the mechanisms underlying the occurrence of symptoms, including LoC, of mTBI, as well as its outcomes. This can be achieved by associating the axonal fiber tracts obtained from tractography with a connectivity atlas, such as the HCP multimodal parcellation (HCP-MMP) (Glasser et al., 2016). However, because the HCP-MMP atlas defines 360 ROIs in the left and right cerebral cortices, numerous axonal fibers would be required to sufficiently describe their connectivity. Although our proposed brain FE model includes 8,602 axonal fibers, the sufficiency of this number is yet to be determined. Moreover, replacing the current group-averaged atlas with subject-specific tractography is expected to introduce variability in local fiber density and orientation dispersion. As discussed above, incorporating such spatial heterogeneity could make the effective anisotropy distribution less uniform. This may result in greater variability of axonal strain across tracts and more focal strain concentrations compared to the present implementation. In addition to inter-subject variability in axonal fiber distributions, individual differences in brain and ventricular geometry may also influence axonal strain by altering the inertial loading of the brain during head rotation. For example, the locations and magnitudes of periventricular strain concentrations could change depending on individual ventricular shapes.

Third, embedding axonal fibers using *CONSTRAINED_BEAM_IN_SOLID in this study led to an approximately six- to eight-fold increase in computational time. In practice, the minimum and maximum wall-clock times were approximately 18 h (case 8, end time 50 ms) and 61 h (case 5, end time 140 ms), respectively. This fact indicates that the proposed model is practically feasible for detailed analyses of a limited number of mTBI cases, whereas its straightforward application to large cohort studies would require further optimization of the model and computational resources. To address the increased computational cost, implementing beam elements only for tracts with higher injury risk could be an effective and practical countermeasure. By contrast, the effectiveness of mass scaling, a technique that increases the mass density of elements within the range that does not affect the result and thereby allows for larger time step sizes and reducing actual computational time, is expected to be limited. This is because that the increase in computational cost was mainly caused by the large number of constraints between beam and solid elements rather than by a reduction in the time step size due to small element dimensions. Therefore, the trade-off between the computational cost and modeling fidelity should be carefully considered when applying the proposed model to large cohort studies. Since the embedded beam elements are modeled with negligible mass and stiffness in this study, axonal strain could in principle be computed in post-processing. However, such an approach would require, for each beam element and each time step, identifying the host solid element, determining its local coordinates, interpolating the strain tensor at that position, and then projecting it onto the beam axis. Given that the present model contains more than 800,000 beam elements, we prioritized an intuitive implementation in which axonal strain is obtained directly during the simulation by specifying a constraint keyword in LS-DYNA, rather than relying on heavy post-processing. Furthermore, the current embedding approach provides a basis for future model extensions, e.g., individual constitutive modeling of axons, glial cells, and their surrounding matrix and the corresponding damage evolution laws, which could allow mechanical interactions between axons and the host tissue to be represented. At present, however, rigorous validation of the individual material properties of these microstructural components remains challenging, as discussed above.

Fourth, the current model limits its representation of the lower brainstem to the level of the medulla oblongata, excluding the spinal cord that continues below it. It remains challenging to accurately represent CSF flow in the spinal canal and to validate the associated ICFD boundary conditions at the foramen magnum. In addition, diffusion MRI-based tractography is known to face technical challenges in reliably reconstructing fiber tracts in the lower brainstem and upper cervical spinal cord, due to limited spatial resolution and pronounced susceptibility- and motion-related artifacts (Zhang and Furst, 2022). Therefore, extending the current model to include a more detailed representation of the lower brainstem and the contiguous spinal cord will require both anatomical modeling efforts and tractography protocols optimized for this region.

Finally, validation and analysis of many brain FE models, including the model proposed in this study, have so far been limited to retrospective approaches, and advanced validation against clinical or neuroimaging outcomes associated with real-world mTBI data remains challenging. To the best of our knowledge, such a comprehensive mTBI research framework is still largely lacking. Key future steps toward prospective validation would include constructing a pre-registered subject database, establishing a subject-specific modeling pipeline that accounts for individual head geometry and tractography, recording and reconstructing head kinematics in real-world mTBI events, and conducting long-term patient follow-up including structural and diffusion MRI and neurocognitive assessments. As mentioned above, it will also be important to relate physical damage in specific axonal tracts to both acute and chronic post-TBI symptoms. This, in turn, will require advanced neurophysiological or network-based models capable of comparing neural activity dynamics before and after injury.

## 5 Conclusion

This study developed a brain FE model that explicitly incorporates axonal fiber tracts obtained from group-averaged tractography data to elucidate the mechanism of mTBI. The validation accuracy of the developed model for brain strain during head impact was quantitatively evaluated using CORA and was found to be better than that of a previous study in two PMHS tests. The model was applied to the reconstruction simulations of eight real-world mTBI cases available in the literature, and the values of injury metrics based on axonal strain were compared for each tract. As a result, in multiple cases where concussion or post-concussion symptoms were reported, higher values of injury metrics were consistently observed at TR_P, CC_B, FAT, CS_P, ML, CPT_P, and SLF. These increases in tract-specific injury metrics can be attributed to the enhanced biofidelity of the brain FE model due to the nonlinear constitutive formulations and ICFD, which facilitated a more precise characterization of axonal strain distribution. In addition, comparisons of the effect of head rotation direction on injury measures for each tract indicated that sagittal rotation contributed the least, whereas horizontal rotation had the greatest contribution. The proposed brain FE model may serve as a foundation for understanding mTBI mechanisms from the perspective of alterations in structural and functional connectomes, as a step toward better mTBI prediction. Future studies will include the following aspects: (1) consideration of microstructures, such as blood vessels, and (2) association of structural damage in specific tracts with disruptions in functional brain networks.

## Data Availability

The raw data supporting the conclusions of this article will be made available by the authors, without undue reservation.
