# Supplementary material for: Axonal tract-integrated finite element brain model for predicting mild traumatic brain injury based on axonal strain
Source: Front Bioeng Biotechnol. 2026 Feb 27;14:1692718. doi: 10.3389/fbioe.2026.1692718 (PMC12982338; doi:10.3389/fbioe.2026.1692718)
Supplement: Supplementary file 1 [file DataSheet1.pdf]

Supplementary Material

1 SUPPLEMENTARY DATA

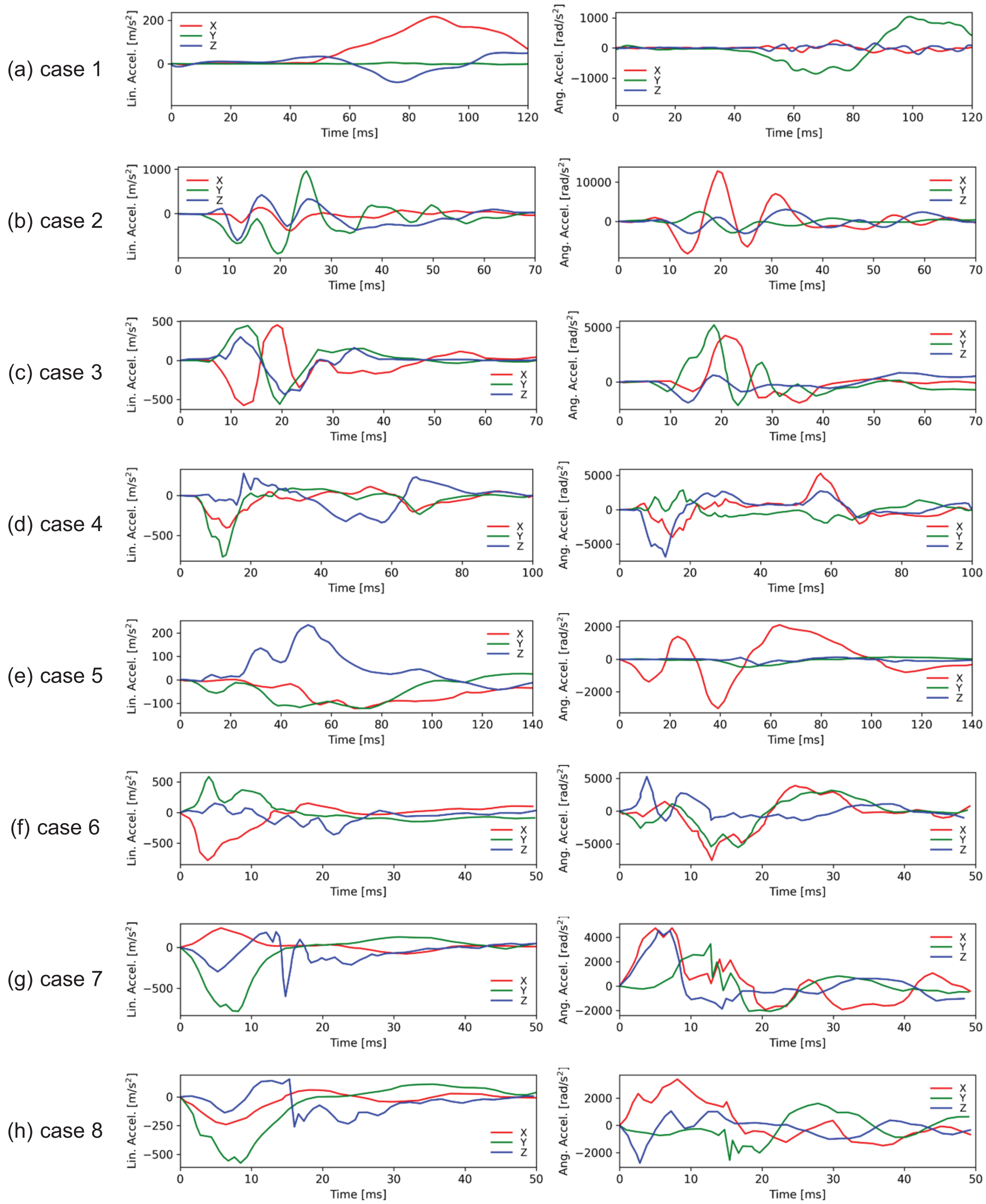

Figure S1. Linear (left column) and angular (right column) acceleration curves inputted to the brain FE model for each mTBI case (Krafft et al., 1998; Atsumi et al., 2020; Hernandez et al., 2015; Zhou et al., 2021; Sanchez et al., 2019; Franklyn et al., 2005; Zimmerman et al., 2022).

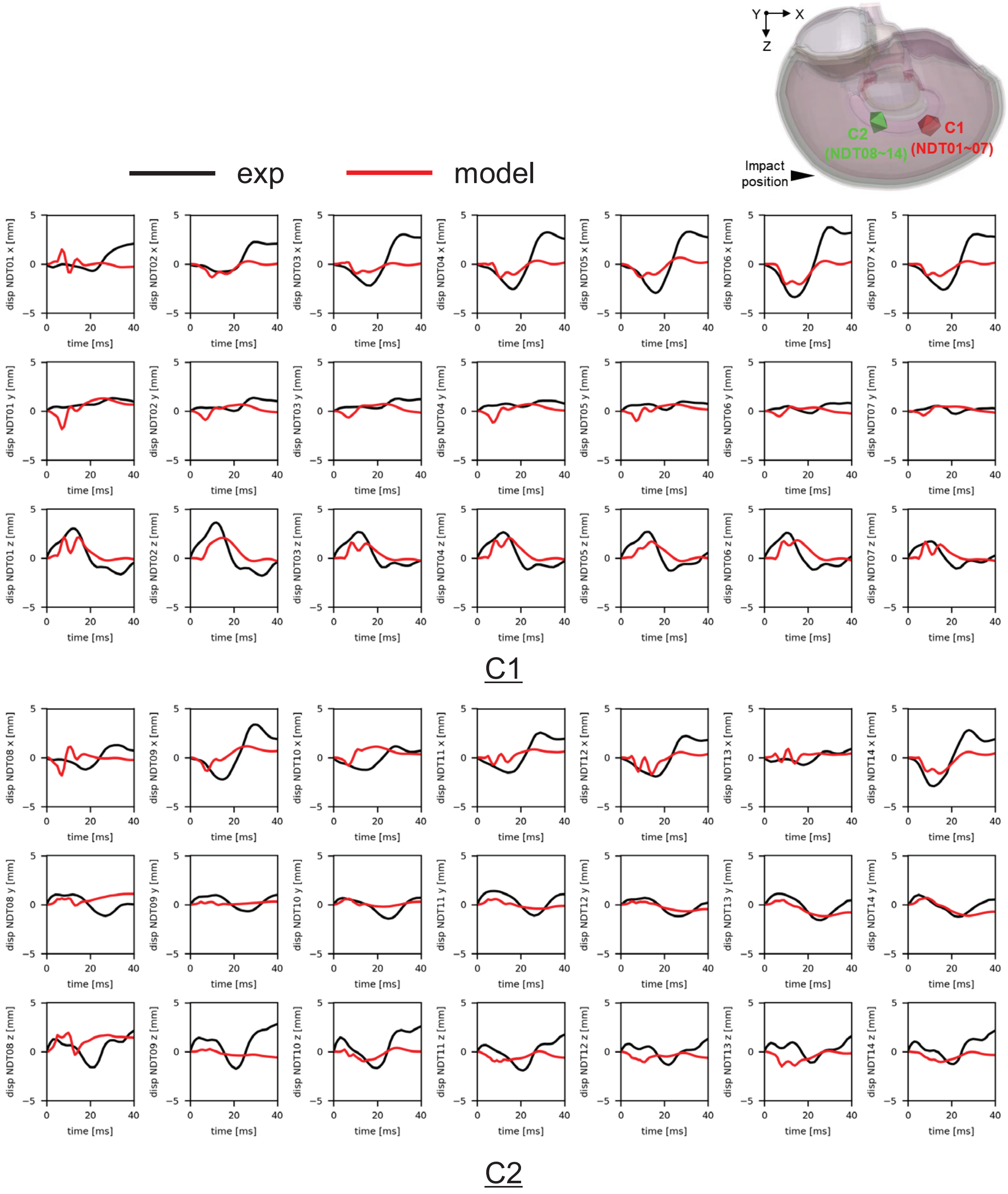

Figure S2. Comparisons of the histories of displacements in NDTs belonging to C1 (upper) and C2 (lower) for C288-T3 between the PMHS test data Hardy et al. (2007) and the simulation results.

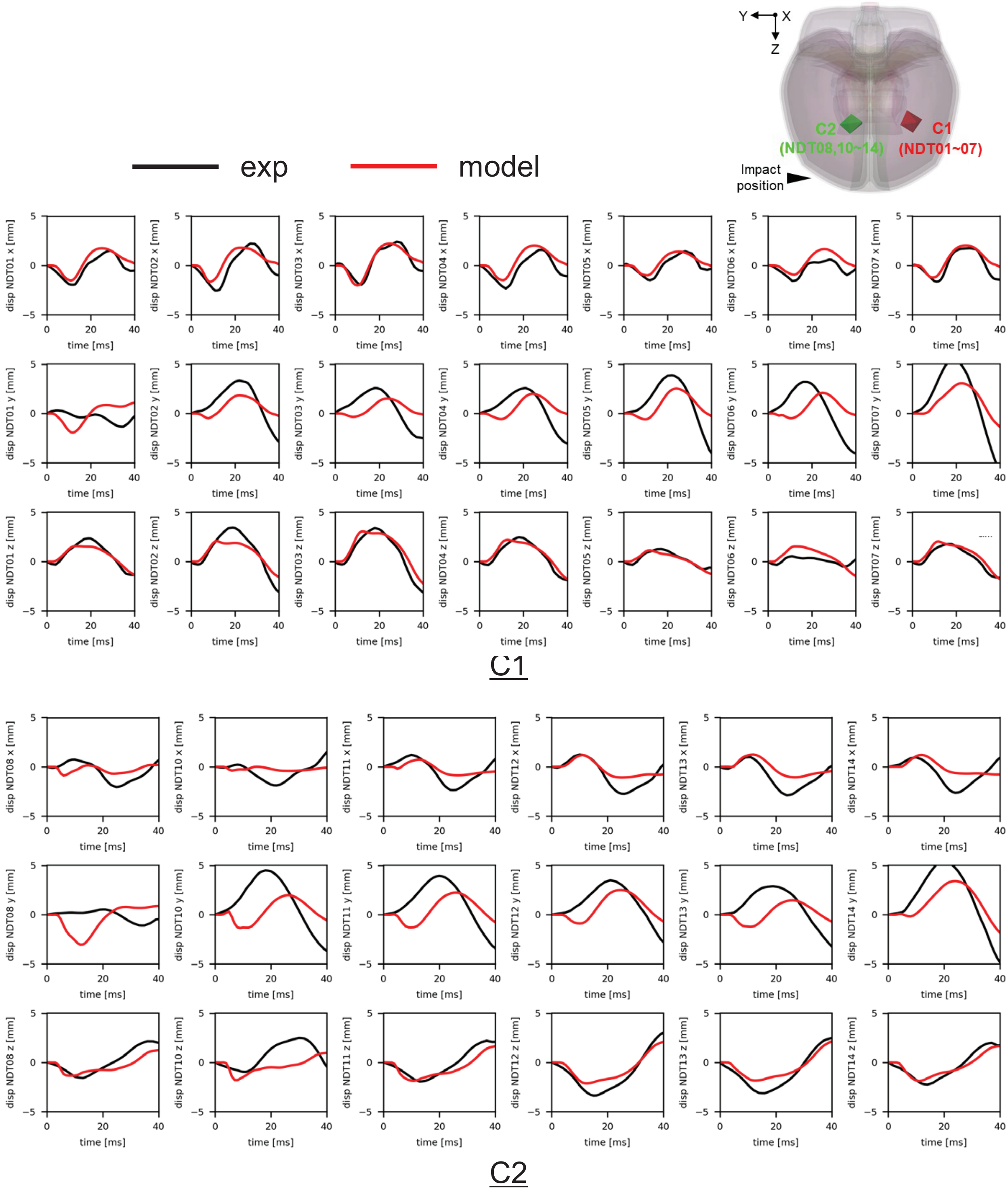

Figure S3. Comparisons of the histories of displacements in NDTs belonging to C1 (upper) and C2 (lower) for C380-T1 between the PMHS test data Hardy et al. (2007) and the simulation results.

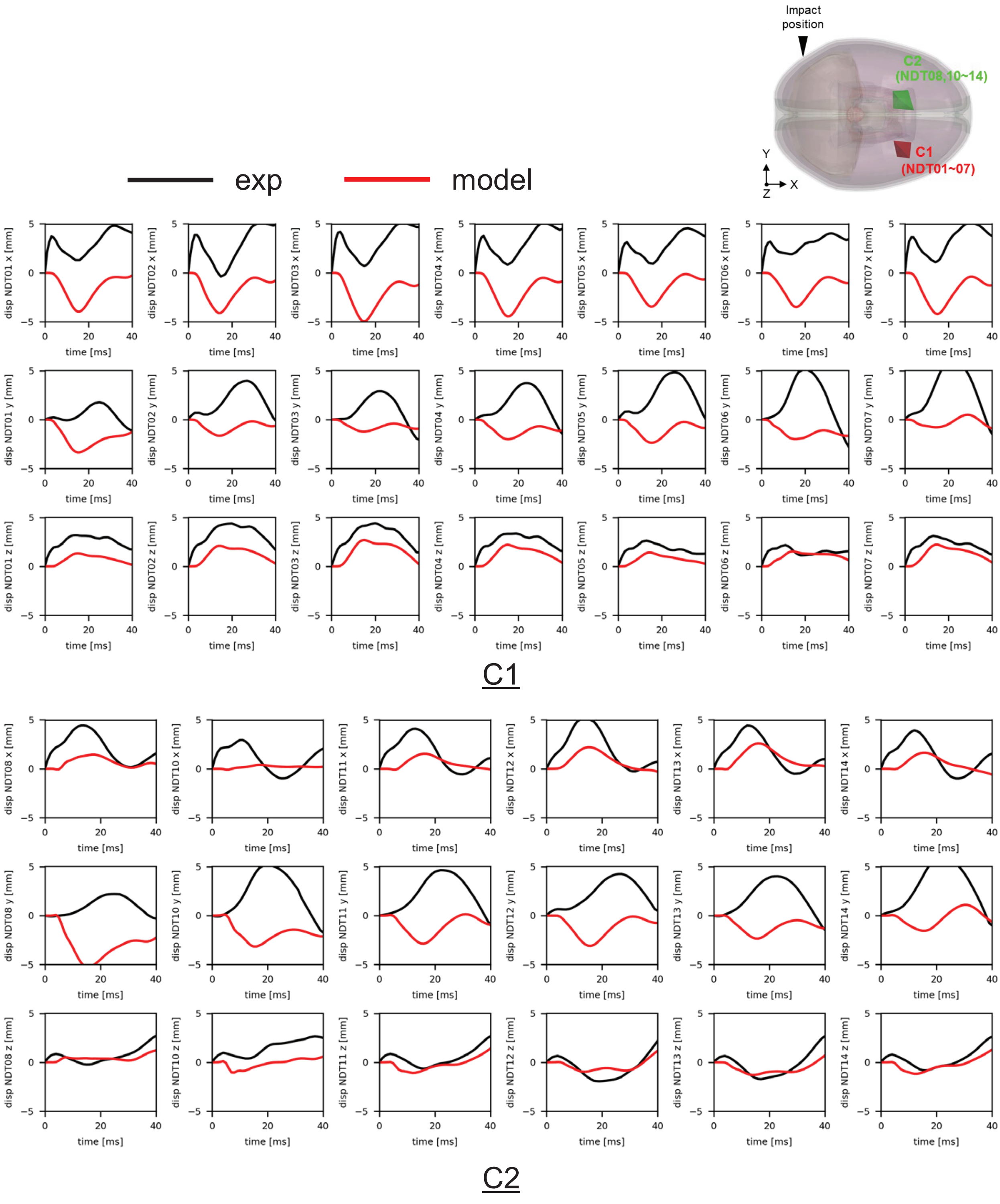

Figure S4. Comparisons of the histories of displacements in NDTs belonging to C1 (upper) and C2 (lower) for C380-T2 between the PMHS test data Hardy et al. (2007) and the simulation results.

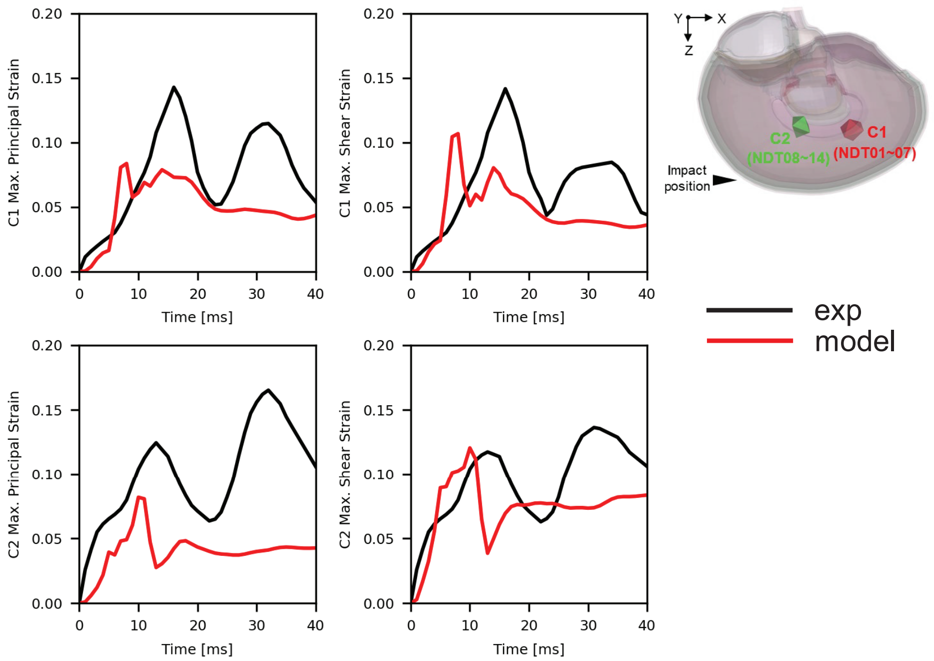

Figure S5. Comparisons of the histories of MPS (left column) and MSS (right column) in C1 (upper row) and C2 (lower row) for C288-T3 between the PMHS test data Hardy et al. (2007); Zhou et al. (2019) and the simulation results. MPS: Maximum principal strain, MSS: Maximum shear strain.

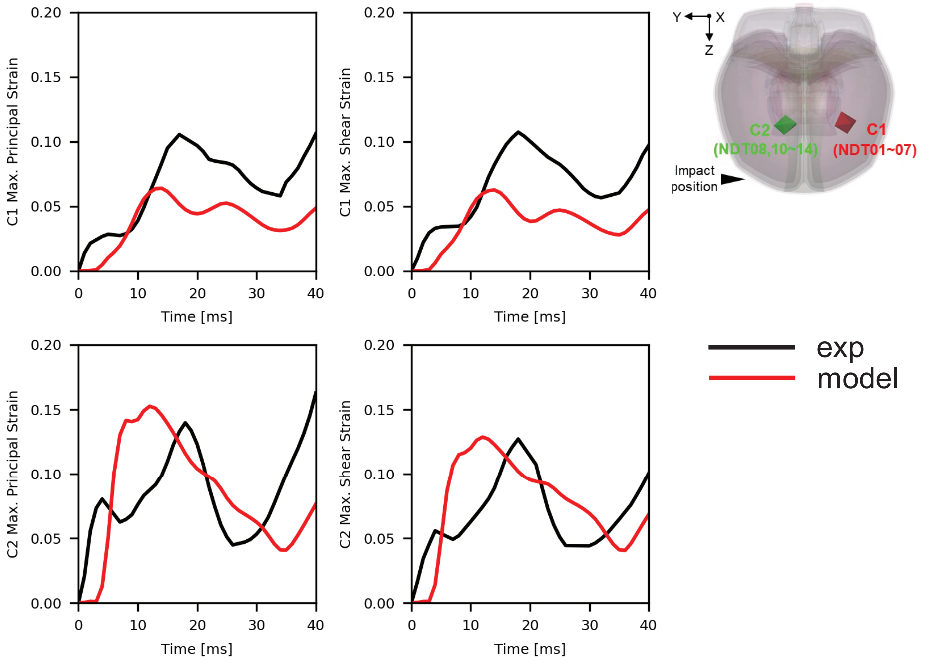

Figure S6. Comparisons of the histories of MPS (left column) and MSS (right column) in C1 (upper row) and C2 (lower row) for C380-T1 between the PMHS test data Hardy et al. (2007); Zhou et al. (2019) and the simulation results. MPS: Maximum principal strain, MSS: Maximum shear strain.

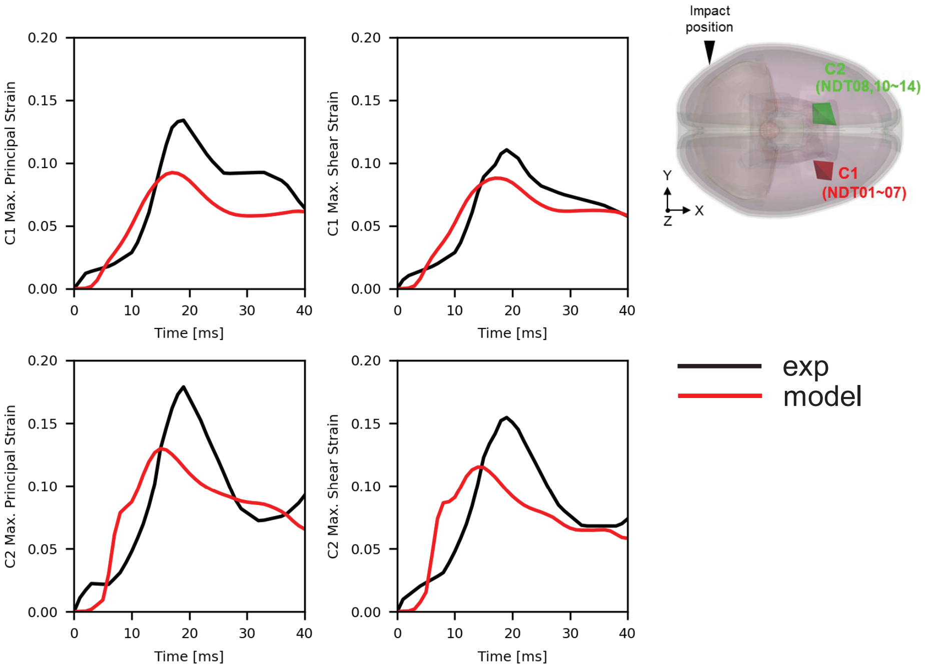

Figure S7. Comparisons of the histories of MPS (left column) and MSS (right column) in C1 (upper row) and C2 (lower row) for C380-T2 between the PMHS test data Hardy et al. (2007); Zhou et al. (2019) and the simulation results. MPS: Maximum principal strain, MSS: Maximum shear strain.

REFERENCES

Atsumi, N., Iwamoto, M., Nakahira, Y., Asano, Y., and Shinoda, J. (2020). Investigation of dynamic deformation of the midbrain in rear-end collision using human brain FE model. *Computer Methods in Biomechanics and Biomedical Engineering* 23, 1236–1246. doi:10.1080/10255842.2020.1795142

Franklyn, M., Fildes, B., Zhang, L., Yang, K., and Sparke, L. (2005). Analysis of Finite Element Models for Head Injury Investigation: Reconstruction of Four Real-World Impacts. *Stapp Car Crash Journal* 49, 1–32. doi:10.4271/2005-22-0001

Hardy, W. N., Mason, M. J., Foster, C. D., Shah, C. S., Kopacz, J. M., Yang, K. H., et al. (2007). A study of the response of the human cadaver head to impact. *Stapp Car Crash Journal* 51, 17–80

Hernandez, F., Wu, L. C., Yip, M. C., Laksari, K., Hoffman, A. R., Lopez, J. R., et al. (2015). Six Degree-of-Freedom Measurements of Human Mild Traumatic Brain Injury. *Annals of Biomedical Engineering* 43, 1918–1934. doi:10.1007/s10439-014-1212-4

Krafft, M., Kullgren, A., and Tingvall, C. (1998). Crash pulse recorders in rear impacts-real life data. *Proceedings of the 16th ESV* , 98–S6–O–10

Sanchez, E. J., Gabler, L. F., Good, A. B., Funk, J. R., Crandall, J. R., and Panzer, M. B. (2019). A reanalysis of football impact reconstructions for head kinematics and finite element modeling. *Clinical Biomechanics* 64, 82–89. doi:10.1016/j.clinbiomech.2018.02.019

Zhou, Z., Domel, A. G., Li, X., Grant, G., Kleiven, S., Camarillo, D., et al. (2021). White Matter Tract-Oriented Deformation Is Dependent on Real-Time Axonal Fiber Orientation. *Journal of Neurotrauma* 38, 1730–1745. doi:10.1089/neu.2020.7412

Zhou, Z., Li, X., Kleiven, S., and Hardy, W. N. (2019). Brain Strain from Motion of Sparse Markers. *Stapp Car Crash Journal* 63, 1–27. doi:10.4271/2019-22-0001

Zimmerman, K. A., Cournoyer, J., Lai, H., Snider, S. B., Fischer, D., Kemp, S., et al. (2022). The biomechanical signature of loss of consciousness: Computational modelling of elite athlete head injuries. *Brain* 146, 3063–3078. doi:10.1093/brain/awac485
